# Indoor Air Pollution and the Health of Vulnerable Groups: A Systematic Review Focused on Particulate Matter (PM), Volatile Organic Compounds (VOCs) and Their Effects on Children and People with Pre-Existing Lung Disease

**DOI:** 10.3390/ijerph19148752

**Published:** 2022-07-19

**Authors:** Tun Z. Maung, Jack E. Bishop, Eleanor Holt, Alice M. Turner, Christian Pfrang

**Affiliations:** 1 UHB NHS Foundation Trust, Inflammation and Aging, University of Birmingham, Edgbaston, Birmingham B15 2TT, UK; tzm031@student.bham.ac.uk; 2 School of Geography, Earth & Environmental Sciences, University of Birmingham, Edgbaston, Birmingham B15 2TT, UK; jack.bishop@liverpool.ac.uk (J.E.B.); e.holt@bham.ac.uk (E.H.); 3 UHB NHS Foundation Trust, Institute of Applied Health Research, University of Birmingham, Edgbaston, Birmingham B15 2TT, UK; a.m.turner@bham.ac.uk

**Keywords:** PM, VOCs, vulnerable groups, indoor air quality

## Abstract

Air pollution affects health, but much of the focus to this point has been on outdoor air. Higher indoor pollution is anticipated due to increasingly energy-efficient and less leaky buildings together with more indoor activities. Studies of indoor air pollution focusing on children and people with respiratory disease from the database Web of Science (1991–2021) were systemically reviewed according to the PRISMA guidelines, with 69 studies included in the final selection. Emissions from building materials affected indoor air quality, and ventilation also had an influence. The main indoor air pollutants are Volatile Organic Compounds (VOCs) and Particulate Matter (PM). PM sources included smoking, cooking, heating, candles, and insecticides, whereas sources of coarse particles were pets, housework and human movements. VOC sources included household products, cleaning agents, glue, personal care products, building materials and vehicle emissions. Formaldehyde levels were particularly high in new houses. Personal exposure related to both indoor and outdoor pollutant levels, highlighting home characteristics and air exchange rates as important factors. Temperature, humidity, educational level, air purifiers and time near sources were also related to personal exposure. There was an association between PM and Fractional exhaled Nitric Oxide (FeNO), lung function, oxygen saturation, childhood asthma and symptoms of chronic obstructive pulmonary disease (COPD) patients. High VOCs were associated with upper airways and asthma symptoms and cancer. Effective interventional studies for PM in the future might focus on human behavior together with air purifiers and increased ventilation, whereas VOC interventions might center more on building materials and household products, alongside purification and ventilation.

## 1. Introduction

The top three causes of death worldwide according to a World Health Organization (WHO) report are cardiovascular, respiratory and neonatal conditions. chronic obstructive pulmonary disease (COPD) was the third leading cause of death globally in 2000 and contributed to 6% of all deaths, with lower respiratory tract infection being the fourth leading cause of death. If all lung diseases such as COPD, lower respiratory diseases, lung cancer and tuberculosis are combined, it becomes the leading global cause of death [1]. Air pollutants, dust, chemicals and socioeconomic status have all been associated with the development, flare-ups and/or progressions of lung diseases, such as asthma and COPD [2].

Due to the rapid development of technology, urbanization and increased population, air pollution has become a hot topic, in particular because of the effects on health. However, much of the focus has been on outdoor air pollution, which is anticipated to decrease in the coming years if public health interventions have their desired effect [3]. One effect of reducing outdoor pollution is likely to be that indoor air pollution will make an increasing contribution to human exposure, due also to increasingly energy-efficient buildings with less ventilation and more indoor activities overall. However, there are many gaps in our understanding of where, when, and how people are exposed to peak concentrations of pollutants in indoor environments [3,4].

**Figure 1:**
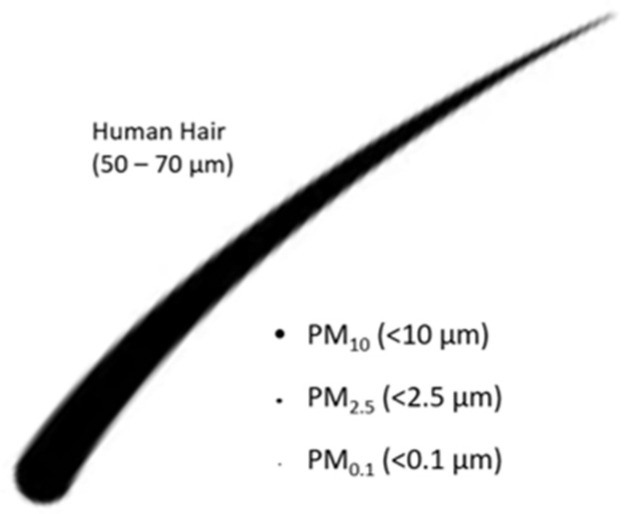
Illustration of the most important size classes of Particulate Matter (PM).

Some of the most important sources of indoor air pollution are Volatile Organic Compounds (VOCs) and Particulate Matter (PM). There are a variety of VOCs emitted from modern household products (e.g., paints, lacquers, cleaning liquids, furnishings, copiers, printers, glues, adhesives or permanent markers). These include non-methane hydrocarbons, halocarbons, benzene, toluene, ethylbenzene, meta-, para- and ortho-xylenes and oxygenated VOCs. There is evidence that these compounds affect human health; for instance, benzene increases the risk of cancer [5]. In addition, multiple outdoor air pollution studies have shown that PM can also affect the health of people [6]. PM is a mix of very small particles and liquid droplets consisting of acids, organic chemicals, metals and dust particles [7], and is typically described by particle size: in particular, PM_0.1_ (droplets or particles of less than 0.1 microns; also referred to as ultrafine particles, UFP), PM_2.5_ (<2.5 microns; fine) and PM_10_ (<10 microns; coarse) based on their aerodynamic equivalent diameters (see Figure 1). Particles greater than 10 microns may be natural (e.g., volcanoes, dust storms) or man-made (e.g., construction), and are mostly filtered out in the nose and airway [8]. Currently, fine particles (PM_2.5_) are most prominent in respiratory health research, but smaller sizes, in particular ultrafine particles (PM_0.1_), may cause more toxicities as they penetrate cell membranes [9].

Some people are more prone to the ill effects of pollution, and these may be termed vulnerable groups (VGs), such as children (age 0–16) [10] and people with pre-existing respiratory disease. Children’s immune and respiratory systems are still developing such that they are vulnerable to exposure to airborne environmental pollutants. People with existing lung problems may have greater sensitivity to pollutants or less reserve to cope with ill effects. Air pollution reduces the life expectancy of VGs by an average of several months (ranging from three days to 11.5 years) [11], and a single exposure can exacerbate diseases of VGs within hours or days [12]. Indoor air pollutants are potentially the most relevant for VGs as they spend particularly long periods of time indoors. Indoor exposures may also vary with demographic factors associated with poor lung health; for instance, children who live in houses with poor ventilation experience more polluted air than present outside [10]. Since a systematic review of outdoor exposures with respect to respiratory health [13] has already been completed, we chose here to systematically review studies of indoor air pollution in VGs to understand how best to focus new studies and design interventions for prevention of future exposure.

## 2. Methods

This systematic review was carried out according to the PRISMA guidelines [14].

### 2.1. Search Strategy

**Figure 2:**
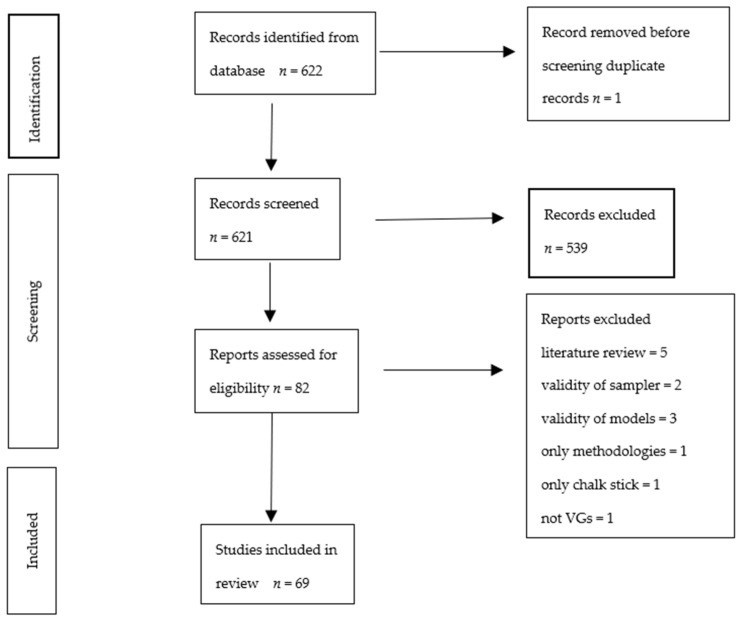
Preferred Reporting Items for Systematic reviews and Meta-Analyses (PRISMA) flow chart of study selection and inclusion.

The Web of Science Core Collection was searched for the years 1991 to 2021 using the search strategy shown in Figure 2, including studies in any language. Eligible articles are summarized in Table 1.

82 studies were available from the literature search, as shown in Figure 2, when focusing the search terms for personal exposure of the selected VGs on our target locations homes, nurseries, hospitals and transport spaces as well as on our target pollutants, VOCs, UFP, PM_1_, and PM_2.5_, in relation to pre-existing conditions/COPD; of these 82 studies, 13 were excluded based on either article type or inaccurate methods regarding air quality measurement or modelling. Sixty-nine studies were thus included in the final review.

### 2.2. Study Selection and Quality Assessment

Initial study selection was carried out by two independent reviewers (JB and CP). If there was disagreement, a referral was made to a third reviewer. Initially, the studies were screened by title and abstract. Subsequently, the full text was read against the inclusion and exclusion criteria. The studies were then divided into groups of VOCs, UFP, PM_1_, PM_2.5_ and PM_10_ (while PM_10_ was not one of our search terms, many studies reporting results on the PM classes of our focus also reported closely related PM_10_ findings that we included in the discussion if relevant). Quality assessment of the studies was carried out by two authors (TZM and EH) according to the Joanna Briggs Institute Critical Appraisal Tool. The initial draft of this review was written by TZM with subsequent input from all co-authors.

## 3. Results

### 3.1. Main Studies Characteristics

Studies originated from around the world with 23 studies from Asia, 22 studies from Europe, 20 studies from North America, three studies from the southcentral Americas and one study from Africa. There were two systematic reviews and two randomized control trials, while the others were observational (cohort, case-control or cross-sectional) studies. Various samplers were used to measure indoor air pollutant levels and personal exposure. Characteristics of the studies are summarized in Table 2.

### 3.2. Study Quality

The studies were generally of low risk of bias. They were of high quality, with omissions most common in areas of identification of co-founding factors. The studies showed both positive and negative correlations with lung disease. However, the literature was slightly biased towards positive studies. Further details are provided in the Supplementary Materials (Tables S1 and S2).

### 3.3. Main Findings

Emissions from building materials of various types were found to affect indoor air quality significantly and ventilation also had a major influence [48]. Studies reported a range of relatively well-defined sources, but they were less clear on the health effects.

#### 3.3.1. Particulate Matter (PM)

##### Sources

Household and transport environments were both found to contain abundant black carbon and UFP [79]. Sources of PM_2.5_ were identified to be smoking, cooking, heating, candles, and insecticides, whereas sources of coarse particles were pets, housework and peoples’ movements [6]. One study identified the main source of polycyclic aromatic hydrocarbons to be the combustion of coal and gasoline for heating [49]. The concentration of black carbon was also noted to be high during the use of charcoal grills and candles [30].

The composition of particulate matter was found to depend on transport modes; iron was mostly found in studies of PM around railways, likely due to the friction of rail wheels and brakes. Zinc and copper were associated with car and bus travel due to particles from brake and tire wear [54]. PM composed of organic and elemental carbon was also found near traffic, likely because organic and elemental carbon has adhesive properties and sticks to coarse particles such as those described above. The high mineral concentration of PM in schools, more so than in homes, was associated with proximity to busy roads and high human occupancy levels [55]. All of these findings highlight the key environmental influences for indoor air pollution.

Personal exposure depended on indoor contact with animals, mold, cooking activities and aerosol use [25], and was also seasonal, such that in winter, indoor levels of PM_2.5_ and PM_10_ were the highest. This is likely due to an increased usage of heaters together with poorer ventilation of houses in winter as people tend to keep windows closed to stay warm. Personal exposure to these particles will thus also increase. Personal exposure also related significantly to indoor and outdoor pollutant levels, which highlighted home characteristics and air exchange rates as important factors for personal exposures. In addition, environmental temperature, humidity, educational level, usage of air purifiers, time near sources and concentration of black carbon also influenced the level of personal exposure [60,61,80].

Children in urban and suburban areas had the highest exposures to UFP in contrast to children in rural areas [58]. Children are exposed to PM more in schools than homes, likely related to the number of people present indoors, in addition to outdoor infiltration [55]. UFP is highest during eating and cooking activities [57]. PM_2.5_ and PM_10_ concentrations in schools are more than double those at home, which highlights the importance of cleaning activities and the density of occupation [36]. Canteen environments have the highest UFP level, whereas libraries have the lowest, which shows the association with cooking and the number of occupants [67].

Exposure to PM depends on the height of the buildings as well. The level of exposure is low in children who study on higher floors, which is likely due to good ventilation [78]. Furthermore, there is infiltration of outdoor PM to indoor, which is compounded by indoor smoking [53] and the usage of mosquito repellents, which cause more indoor exposure.

Associations between indoor and outdoor PM are stronger in schools near main or small roads than for those away from traffic. PM_2.5_ and the number of particles is high during rush hour traffic but sometimes reach their peak in relation to human activities such as smoking and using mowers. The indoor number of particles is occasionally affected by cooking, cleaning and floor polishing, which illustrates that human activities lead to high levels of these particles [39]. Participants spend 85% of their time indoors and the highest indoor exposure to UFP is reached during sleeping, highlighting the importance of controlling indoor air pollution [75]. Air filtration can significantly lower the PM level in houses with smokers, which is promising for future studies [17].

##### Health Effects

There is an association between Fractional exhaled Nitric Oxide (FeNO) and PM_2.5_ and PM_10_ exposure in asthma patients, with a significant increase in FeNO levels in exposed asthma patients. The strongest association was found between FeNO and two-day average PM concentration. The association of elemental carbon and NO_2_ with asthma was weak [32]. However, there was no association between asthma and spirometry, oxygen saturation, heart rate, or blood pressure [44].

When it comes to PM_2.5_ exposure and force expiratory volume in one second (FEV1), peak expiratory flow rate (PEFR) and maximal mid-expiratory flow (MMEF), it was found that FEV1 reduction occurred with exposure to PM_2.5_ in adult COPD patients. In asthmatic children not taking inhaled corticosteroids or montelukast, a drop in FEV1, PEFR and MMEF was noted [68]. There was a significant reduction in PEFR and a rise in symptoms such as cough and sputum when COPD patients were exposed to PM_2.5_ [26].

In a randomized cross-over study, there was a relationship between lung function test and lung-deposited particle surface area concentration (PSC), size-specific particle number concentration (PNC), and particle mass concentration (PMC) of PM_1_, PM_2.5_ and PM_10_ from candles burning, the toasting of bread and the frying of sausages. PMC from candle burning and frying sausages and PM_2.5_ and PSC from candle burning decreased lung function, but PMC from toasting bread and the PNC of UFP were not associated with lung function changes [66].

Short-term exposure to PM caused an acute decline in blood oxygen saturation which was most obvious in the first three hours but became less obvious after three hours in both COPD patients and healthy people. However, the reduction in blood oxygen saturation was more significant in COPD patients than in healthy subjects [74].

There was a relationship between childhood asthma and women exposed to PM_2.5_, and black carbon and nicotine during pregnancy [77]. In addition, black carbon from combustion is strongly associated with high systolic blood pressure [22].

#### 3.3.2. Volatile Organic Compounds (VOCs)

##### Sources

The sources of VOCs reported in the included studies were household products, cleaning agents, glue, personal care products, building materials, solvents, smoking and vehicle emissions. Formaldehyde levels were particularly high in new houses with new furniture [6,69,70]. The four most reported VOCs were toluene, m-/p-xylene, alpha-pinene and delta-limonene [63]. Toluene is the most abundant aromatic hydrocarbon [70]. N-hexane, 1,1,1-trichloroethane, benzene, toluene, ethylbenzene, m-, p-xylene, dodecane and hexadecane are hazardous air pollutants that cause cancer as well as eye and skin irritation [18].

Most VOCs come from indoor sources in urban, semirural and residential areas, but there was an outdoor influence in industrial areas. Alkanes and aromatic compounds were found in all areas with variable chemical distributions. C9-C11 alkanes, toluene and xylenes were mostly found indoors, contributed to by human activities such as renovations, painting and cleaning. Hexane, heptane and benzene dominated outdoor industrial areas but also influenced indoor air [50]. High levels of o-xylene and ethylbenzene were identified in winter [51]; formaldehyde, acetaldehyde and toluene concentrations were found to be the highest in bedrooms [35]. One study reported differences in the concentrations of specific VOCs between the ground floor and basement levels (e.g., higher amounts of nonanal and 2-butoxyethanol at basement levels, but more naphthalene and 2-ethylhexan-1-ol at ground floor levels), likely because of the extensive use and storage of household cleaning products, deodorizers and solvents at both ground floor and basement levels in these study locations, and emissions from vehicles more easily infiltrating into the ground floor and basement levels compared with higher floors [70]. Previous works have found that VOC levels in basements often exceeded those on ground floor living spaces because basements in residential locations are commonly used for chemical storage. Interestingly, VOCs, CO, and CO_2_ were not linked to cooking, unlike PM_10_ and PM_2.5_ [29].

Socioeconomic factors influenced the personal exposure to VOCs; for example, parental education, age, and type of housing had a slight contribution [18].

##### Health Effects

High VOC and CO levels were associated with worsening asthma symptoms [28]. Children living close to industrial sites had more exposure to VOCs, and there was a significant association with school absence because of sore throat, cough and cold. O-xylene emitted from industrial activity had a clear association with respiratory symptoms [27]. Lifetime cancer risk was associated with benzene exposure [50]. There is an association between VOCs and childhood acute leukemia. Benzene, in particular, is a relevant carcinogen causing leukemia [40].

## 4. Discussion

We found an abundance of studies describing potential sources of indoor air pollution; however, studies of the health effects were comparatively less common and may be a fruitful area for future research.

The sources of PM were mainly related to the burning of materials, friction of metals in transportation and cleaning activities spreading these particles. PM is thus particularly abundant near stoves and transportation. Personal exposure to these particles depends on the duration of time people spend near these sources. The density of occupation and degree or type of cleaning activities appeared to influence PM exposure markedly, as shown by the higher personal exposure in schools and canteens compared to private homes. This highlighted that people’s movements and activities spread the particles, thereby causing elevated personal exposures. Furthermore, indoor PM levels were high in winter, which is likely due to less frequent ventilation and the usage of heaters, and personal exposure was affected proportionately.

In addition, the composition of PM, such as iron near subways, zinc, and copper near cars and buses highlights the environment’s influence on indoor PM. Children in rural areas have less personal exposure than their counterparts in urban areas, which again highlights the influence of the environment on indoor air pollution. The ventilation of houses also strongly affects the indoor pollutant levels. This should be considered in future housing projects when choosing the appropriate locations and designs for housing. More studies are required to investigate how good quality housing can reduce personal exposure. In one RCT, air purifiers reduced levels of personal exposure, but many more studies are needed to prove this hypothesis. If it is proven to work, air purification could become one of the key mitigation strategies to tackle indoor air pollution. Studies looking into the usage of air purifiers to improve airway problems in VGs should be particularly encouraged.

Human activities, behavior and education level are associated with personal exposure to air pollutants. However, there is no study looking into the level of personal exposure vs change of behavior (e.g., changing cooking stoves from open fire to conventional gas/electric or induction hob). There is a clustered RCT about cookstove interventions to improve infant health in Ghana. Liquefied petroleum gas (LPG) cookstoves or improved biomass cookstoves were introduced for pregnant women to see if there was any improvement in infant health. The studies resulted in no improvement in birth weight and in the risk of severe pneumonia in the first 12 months. The researchers concluded that the effect could be due to a lower-than-expected reduction of air pollutants [82]. The effects of changing the human behavior of VGs on indoor air pollution and health should be investigated in the future.

A few studies have investigated the association between upper airway symptoms, lung function test, PEFR, FeNO and oxygen saturation with personal exposure to PM. This is consistent with PM causing inflammation in the airways affecting lung function and FeNO. Stronger evidence is required to draw firm conclusions on this topic. Similarly, larger studies are needed to prove the link between PM and cardiovascular diseases such as high blood pressure and coronary artery disease.

VOCs are mainly related to household products, home renovations, smoking, and the use of solvents. Therefore, VOC levels are high in the areas where these products are extensively used and stored, especially on the ground floor or in basements. Emissions from vehicles infiltrate ground floor and basement levels more easily than high floors, which compounded VOC levels both on ground floors and in basements. There is also a seasonal variation of VOC levels. They are generally high in winter and are likely due to the poor ventilation of homes in winter. Although the main sources of VOCs are indoors, there is some outdoor influence in industrial areas. Socioeconomic factors and level of education have also an influence on personal exposure to VOCs. Interestingly, VOC exposure is not related to cooking.

VOC exposure appears to irritate the airways, causing upper airway symptoms such as the common cold, cough and sore throat. It also increases asthma exacerbation rates likely due to a similar mechanism. There is an association between VOCs and cancer, and benzene specifically increases the risk of leukemia, but stronger evidence is needed. Interventions to change human behavior and the use of indoor air purifiers should be a focus in the future.

### Strength and Limitations

The articles in question were searched extensively using an appropriate search strategy from a large database without limitation to language and reviewed systematically. A quality check was performed using the Joanna Briggs Institute Critical Appraisal Tool. It should be noted that the chosen database (Web of Science) focusses on peer-reviewed literature and will not generally include work released e.g., as grey literature. The search terms also did not encompass all potential VGs.

## 5. Conclusions

Indoor air pollution sources are varied, with significant differences seen between urban and rural areas and between public locations (e.g., schools) and private homes, the latter being less well studied. PM are mainly associated with cooking, heating and metal frictions, whereas VOCs are mostly associated with household products, personal care products and building materials. The exposure route is mainly the respiratory tract, as these particles and volatile compounds mostly enter the body via inhalation. Other possible exposure routes are via the skin and eyes [18]. The effects on health are likely, based on a small number of relevant studies together with extrapolation from the outdoor air pollution literature, but are relatively poorly reported. It is very likely that VOCs cause upper airway irritation and that PM causes inflammation of the airways affecting lung function and FeNO. Interventional studies for PM in the future might focus on reducing sources related to human behavior together with air purifiers and increased ventilation, whereas VOC studies might need to center more on building materials and air purification and ventilation.

## Figures and Tables

**Table 1:** Overview of studies eligible for inclusion in the systematic review.

Study Design	Participant	Environment	Pollutant Outcome	Health Outcome
Systematic review, Randomized Controlled Trial (RCT), observational studies	Children; people with pre-existing conditions (e.g., asthma and COPD patients)	Personal exposure in homes, schools, nurseries and hospitals	VOCs, UFP, PM_1_ and PM_2.5_	Symptoms Lung function Quality of life

**Table 2:** Overview of the characteristics of the studies included (in alphabetical order of the first author of each study).

Author/Reference	Country	Study Type/Design	Number of Participants and Their Characteristics	Methods of Indoor Pollution Assessments and Collection Time	Pollutant Analysis (Including Indoor-Outdoor)	Method of Health Effect Measurement	Results
Adgate, J. L., et al., (2004) [15]	USA	Prospective cohort	Children from 2 inner city schools	Organic vapor monitors, 1999, 2000.	VOCs		Home had largest and the school and outdoor environments had the smallest influence on personal exposure to VOCs.
Adgate, J. L., et al., (2004) another article [16]	USA	Prospective cohort	Children from 284 house holds	Organic vapor monitors, 1997	VOCs		Personal exposure was strongly associated with home indoor environment after controlling for important covariates.
Batterman, S., et al., (2005) [17]	USA	Prospective cohort	4 single family home environments	Four speed HEPA filter unit	PM, VOCs		Air filters can significantly lower PM concentrations in smoker’s homes if air exchange rates are limited.
Byun, H., et al., (2010) [18]	Korea	Prospective cohort	50 children	Organic vapour monitors, 2008	VOCs		Parental education, year of home construction and type of housing were correlated with personal VOC exposure.
Broich, A. V., et al., (2012) [19]	Germany	Prospective cohort	16 participants	Optical aerosol spectrometer and a small video camera, 2010.	UFP, PM_10_, PM_2.5_		Smoking and cooking were the main indoor sources for PM and the personal exposure significantly exceed the outdoor particulate matter concentrations.
Buonanno, G., et al., (2012) [20]	Italy	Prospective cohort	103 children	Hand-held UFP counters equipped with GPS Tracking, 2011, 2012.	UFP		Most of the children exposure take place at home during cooking/eating time at home and time spent in traffic jams.
Buonanno, G., et al., (2013) [21]	Italy	Prospective cohort	103 children	Black carbon monitor, hand-held UFP counters equipped with GPS tracking, 2011, 2012.	UFP and Black carbon (BC)		High levels typically detected in urban traffic microenvironments. Cooking and using transportation were the main daily exposure.
Baumgartner, J., et al., (2014) [22]	China	Prospective cohort study	280 women	Chemical and optical methods	UFP, PM_2.5_, black carbon	Blood pressure	Black carbon from combustion is more strongly associated with blood pressure than PM mass, and that BC’s health effects may be larger among women living near a highway due to greater exposure to vehicle emissions.
Branco, P., et al., (2014) [23]	Portugal	Cross-sectional	3 nurseries	TSI DustTrak DRX 8534 particle monitor, 2013.	PM_1_, PM_2.5_, PM_10_		Indoor sources (re-suspension phenomena due to children’s activities, cleaning, and cooking) were the main contributors to indoor PM concentrations, but poor ventilation of classrooms affected indoor air quality by increasing the PM accumulation.
Beko, G., et al., (2015) [24]	Denmark	Cross-sectional study	60 non-smoking residents	NanoTracer, 2013.	UFP		The home accounted for 50% of the daily personal exposure. Indoor areas other than home or vehicles contributed 40%. The highest median UFP concentration was obtained during passive transport (vehicles).
Cortez-Lugo, M., et al., (2008) [25]	USA	Prospective cohort	38 asthma children and COPD adults	MiniVol sampler, personal pumps, 2000	PM_2.5_ and PM_10_	Effects of PM exposure to lung function in asthma and COPD	Consistent decrements in MMEF in children with asthma who were not receiving medications.
Cortez-Lugo, M., et al., (2015) [26]	Mexico	Prospective cohort	29 adults with COPD	Personal pumps, 2000.	PM_2.5_	Lung function and respiratory symptoms	Exposure to PM_2.5_ was associated with reductions in peak expiratory flow (PEF) and increased respiratory symptoms in adults with COPD.
Cipolla, M., et al., (2016) [27]	Italy	Prospective cohort	74 students	Perkin Elmer Italia S.p.A, 2006.	VOCs	Rates of school absenteeism	The VOC levels were significantly higher in the industrial areas causing absence from school due to sore throat, cough, and cold. O-Xylene is associated with respiratory symptoms.
Cleary, E., et al., (2017) [28]	USA	Cross-sectional	2 cities	E Q-Trak Indoor Air Quality Monitor, Formaldehyde Multimode Monitor, e P-Trak Ultrafine Particle Counter, 2017.	VOCs, PM, CO	Asthma symptoms	Average CO concentrations were high, which is potentially associated with increased asthma symptoms.
Cheung, P. K., et al., (2019) [29]	Hong Kong	Prospective cohort	Seven subdivided units	Portable Aeroqual monitors, 2018.	CO, CO_2_, PM_10_, PM_2.5_ and VOC.		Mean PM_10_ and PM_2.5_ concentrations during cooking were higher than the pre-cooking levels but cooking did not increase CO, CO_2_, and VOC concentrations.
Cunha-Lopes, I., et al., (2019) [30]	Portugal	Prospective cohort	9 children	SKC five-stage Sioutas Cascade Impactor, 2018.	PM_1_, BC, UFP		High peak BC levels in underground parking lots, during charcoal grills, and candles were burning.
Curto, A., et al., (2019) [31]	Mozambique	Prospective cohort	202 women	A high-volume sampler, 2014, 2015	UFP and Black carbon		Main determinants of mean and peak personal exposure to BC were lighting source, kitchen type, ambient EC levels, and temperature.
Delfino, R. J., et al., (2006) [32]	USA	Prospective cohort	48 asthmatic children	Personal PM_2.5_ monitor, Harvard impactor. 2003,2004.	PM_2.5_, NO_2_, Elemental carbon		The strongest positive associations were between FENO and 2-day average pollutant concentrations. Strong associations were found for ambient elemental carbon and weak associations for ambient NO_2._
Diapouli, E., et al., (2007) [33]	Greece	Cross-sectional	7 primary schools	Portable Condensation Particle Counter, cold period of 2003, 2004	UFP		The highest mean indoor concentrations were found in a small carpet-covered library and a teachers’ office. The highest outdoor concentrations were affected by heavy traffic. Indoor-to-outdoor concentration (I/O) ratios were below 1.
Diapouli, E., et al., (2008) [34]	Greece	Cross-sectional	7 primary schools	Harvard PEMs, 2003, 2004	UFP, PM_2.5_, PM_10_		Very high I/O ratios were observed when intense activities took place.
Fang, L., et al., (2019) [35]	China	A double-blind, randomized crossover trial	20 asthma patients	Low-cost pump packages. 2017.	VOCs		Levels of formaldehyde, acetaldehyde, and toluene were highest in the bedrooms. Air cleaners in houses lead to significant reductions in VOC concentrations indoors, but the associated health risks are still of concern.
Faria, T., et al., (2020) [36]	Portugal	Prospective cohort	5 schools, 40 homes, and 4 transportation modes.	Medium volume samplers, light scattering laser photometer. 2017, 2018.	UFP, PM_2.5_, PM_10_	Health effects due to developing immune, respiratory, central nervous, digestive and reproductive systems	Indoor environment is the main contributors to personal exposure to PM.
Gokhale, S., et al., (2008) [37]	Germany	Prospective cohort	7 adults	Organic vapour monitor, 2005	VOCs		The largest contribution of VOCs to the personal exposure is from homes, followed by outdoors, and the offices.
Goyal, R. and M. Khare (2009) [38]	India	Prospective cohort	A three–storied naturally ventilated school	Environmental dust monitor, IAQ monitor, 2006,2007	PM_1_, PM_10_, PM_2.5_		PM concentrations in classroom exceeds the permissible limits and indoor/outdoor levels for all sizes of particulates are greater than 1 and influence of ventilation rate and of traffic was found.
Guo, H., et al., (2010) [39]	Australia	Cross-sectional	A primary school	Two scanning mobility particle sizers, 2006	UFP, PM_2.5_		Early morning and late afternoon peaks of number of particles and PM_2.5_ were observed at traffic rush hours and the temporal variations of those related to human activities such as cigarette smoking and the operation of a mower. The indoor air pollution is affected by the outdoor and influenced by indoor sources, such as cooking, cleaning, and floor polishing activities as well.
Gao, Y., et al., (2014) [40]	China	1:1 matched case control study	105 children with acute leukemia	Diffusive sampler, 2008–2011	VOCs, NO_2_	Association between indoor air pollutants and childhood acute leukemia	High concentrations of NO_2_ and almost half of VOCs were associated with the increased risk of childhood AL.
Garcia-Hernandez, C., et al., (2019) [41]		Systemic review			UFP		The levels of UFP were correlated with heavy traffic or cooking and cleaning activities.
Habil, M. and A. Taneja (2011) [42]	India	Cross-sectional	4 schools	Grimm aerosol dust Monitor, 2007, 2008	PM_1_, PM_10_, PM_2.5_		The average indoor/outdoor ratios were >1 and there was poor correlation.
Hoang, T., et al., (2017) [43]	USA	Cross-sectional	34 early childhood education environments	Q- TRAK™ IAQ Monitors, SKC AirChek 2000 pumps, VOC sampler, 2010, 2011.	VOCs		VOCs found in cleaning and personal care products had the highest indoor concentrations.
Jansen, K. L., et al., (2005) [44]	USA	Prospective cohort	16 asthma or COPD patients	PM_2.5_ and PM_10_ Harvard Impactor, Marple Personal Environmental Monitors for PM_10_, 2002, 2003	PM_2.5_, PM_10_	FeNO, spirometry, exhaled breath condensate, pulse oximetry, heart rate, blood pressure, symptom, and medication use	An increase in outdoor, indoor, and personal black carbon was associated with increases in FENO but no significant association was found in spirometry, blood pressure, pulse rate, or SaO_2_.
Jeong, H. and D. Park (2017) [45]	Korea	Prospective cohort	44 children	Micro-aethalometer, 2015, 2016.	UFP and Black carbon		Diesel vehicles, subway, cooking, and smoking increase BC exposure.
Jeong, H. and D. Park (2018) [46]	Korea	Prospective cohort	40 children	Microaethalometer AE-51, 2015, 2016	black carbon		Transportation and cooking led to frequent peak levels.
Kearney, J., et al., (2011) [47]	Canada	Prospective cohort	45 homes of non-smoking adults and 49 homes of asthmatic children	Portable condensation particle counter, 2005,2006	UFP	.	Outdoor levels generally exceeded indoor levels, but indoor concentrations were higher around 5–7 pm, suggesting a strong influence of cooking. Large indoor peaks and low infiltration of ambient PM resulted in the indoor sources contributing more than infiltrated UFP.
Kalimeri, K. K., et al., (2016) [48]	Greece	Prospective cohort	3 public primary school	Radiello passive samplers, Gammadata RAPIDOS samplers, 2011, 2012	VOCs, NO_2_, Ozone	Possible health risks at school as measured by lifetime cancer risk	Emissions from building materials have a significant contribution to the indoor air quality. The estimated average lifetime cancer risks for benzene, formaldehyde and trichloroethylene were very low.
Liu, Y. W., et al., (2020) [49]	China	Prospective cohort	13 children	Personal sampling pump, 2018, 2019	UFP, PAHs	Lifetime cancer risk	Coal combustion and gasoline were main sources during heating and non-heating seasons. There was significant increase in PAHs and the incremental lifetime cancer risk in the heating season.
Massolo, L., et al., (2010) [50]	Argentina	Prospective cohort	93 school and houses, 33 outdoor areas	Passive 3 M monitor, 2000–2002	VOCs		Most VOCs predominantly originated indoors in urban, semirural, and residential areas, whereas an important outdoor influence in the industrial area was observed.
Mainka, A. and B. Kozielska (2016) [51]	Poland	Prospective cohort	48 children	Perkin Elmer stainless steel tube samplers. 2013, 2014.	VOCs (BTEX)	The health risk as measured by cancer risk	Elevated levels of o-xylene and ethylbenzene were found in all monitored classrooms during the winter season. Outdoor concentrations were lower than indoors. Chronic health effects associated with carcinogenic benzene or non-carcinogenic BTEX were high.
Mazaheri, M., et al., (2014) [52]	Australia	Cross-sectional	137 children	Philips Aerasense Nanotracers (NTs), 2010–2012	UFP		Outdoor activities, eating/cooking at home, and commuting were the three activities causing the highest exposure. Children’s exposure during school hours was more strongly influenced by urban background particles than traffic near the school.
Mazaheri, M., et al., (2019) [53]	China	Prospective cohort	24 children	Philips Aerasense NanoTracers, 2016.	UFP		Indoor exposure was significantly higher than outdoor exposure which was due to smoking and the use of mosquito repellent.
Martins, V., et al., (2020) [54]	Portugal	Cross sectional study	4 homes and 4 schools	Personal Cascade Impactor Sampler. 2017–2018.	UFP		PM chemical composition depended on transport mode. Fe was the component of metro PM, derived from abrasion of rail -wheel -brake interfaces. Zn and Cu in cars and buses PM were related with brake and tyre wear particles.
Martins, V., et al., (2021) [55]	Portugal	Cross sectional study	Assigned bicycle, bus, car and metro route in Lisbon	Personal environmental monitor. 2018	UFP		Black carbon concentrations when travelling by car was higher than in the other transport modes due to the closer proximity to exhaust emissions. Personal exposure to PM_2.5_ is higher in cycling than car due to higher inhalation rate and longer journey time.
Phillips, M. L., et al., (2005) [56]	USA	Prospective cohort	39 participants	Personal sampling pump	VOCs		Personal and indoor concentrations were higher than outdoor concentrations, indicating that indoor exposures were dominated by indoor sources.
Paunescu, A. C., et al., (2017) [57]	Paris	Prospective cohort	96 children	MicroAeth^®^AE51, DiSCmini^®^, 2014, 2015.	UFP and Black carbon		BC exposure was high during trips (principally metro/train and bus), while UFP exposure was high during indoor activities (mainly eating at restaurants).
Pacitto, A., et al., (2020) [58]	Italy	Prospective cohort	60 children	Handheld diffusion charger particle counter, 2018–2019	UFP		Non-school indoor environment causes most children’s exposure.
Raaschou-Nielsen, O., et al., (1997) [59]	Denmark	Cross-sectional	98 children	Diffusive VOC samplers, 1995	VOCs		The front-door concentrations were significantly higher in Copenhagen than in rural areas, but the personal exposures were only slightly higher.
Rojas-Bracho, L., et al., (2000) [60]	USA	Prospective Cohort	18 COPD patients	Modified PM_2.5_ and PM_10_ personal exposure monitor and a single personal pump, 1996, 1997	PM_2.5_, PM_10_		The strength of the personal-outdoor association for PM_2.5_, was strongly related to that for indoor and outdoor levels.
Rojas-Bracho, L., et al., (2004) [61]	USA	Prospective cohort	18 COPD patients	Modified personal exposure monitor, 1996, 1997	PM_2.5_, PM_10_		The relationship between personal PM_2.5_ exposures and the corresponding ambient concentrations was influenced by home air exchange rates.
Rufo, J. C., et al., (2015) [62]	Portugal	Cross-sectional	10 public primary schools	Portable condensation particle counters, 2014	UFP		The average indoor UFP number concentrations were not significantly different from outdoor concentrations. The levels of carbon dioxide were negatively correlated with indoor UFP concentrations. Occupational density was significantly and positively correlated with UFP concentrations.
Shendell, D. G., et al., (2004) [63]	USA	Prospective cohort	7 schools	Organic vapour monitor and DNSH passive aldehydes and ketone sampler, 2001	VOCs		The main sources of aldehydes in classrooms were likely interior finish materials and furnishings made of particleboard without lamination. The four most common VOCs measured were toluene, m-/p-xylene, alpha-pinene, and delta-limonene.
Sexton, K., et al., (2005) [64]	USA	Prospective cohort	150 children	Passive sampler, bloods, and urine sample, 2000, 2001	VOCs		There were strong statistical associations between measured blood VOC concentrations in siblings in the same household.
Sohn, H. and K. Lee (2010) [65]	Korea	Prospective cohort	2 vehicles	Portable aerosol spectrometers	UFP, PM_2.5_		A single cigarette being smoked could exceed the US EPA NAAQS of PM under realistic window opening conditions.
Soppa, V. J., et al., (2014) [66]	Germany	randomized cross-over controlled exposure study	55 healthy volunteers	Fast Mobility Particle Sizer, Aerodynamic Particle Sizer, Nanoparticle Surface Area Monitor	PM_1_, PM_10_, PM_2.5_	Respiratory health as measured by lung function	High levels of indoor fine particles from certain sources may be associated with small decreases in lung function in healthy adults.
Slezakova, K., et al., (2019) [67]	Portugal	Cross-sectional	20 public primary schools	Portable condensation particle counters. 2014, 2015.	UFP		Outdoor emissions contributed to indoor UFP. Canteens had the highest UFP levels. Cooking on school grounds caused elevated UFP in the classrooms. Lowest UFP were found in libraries mostly due to the limited occupancies.
Trenga, C. A., et al., (2006) [68]	USA	Prospective cohort	57 elderly, 17 children	Harvard impactor, personal monitor. 1999–2001.	PM_2.5_, PM_10_	Lung function changes to daily indoor, outdoor, and personal PM	Maximal midexpiratory flow (MMEF) was decreased in children with asthma who were not receiving medications. The effects were observed even though PM exposures were low for an urban area.
Tran, T. D., et al., (2020) [69]	Vietnam	Cross-sectional	10 nursery schools	Adjustable mini air Samplers, 2017, 2018	BTEX	Health risk as measured by life-time cancer risk	Outdoor BTEX originated from the common sources, which consisted mainly of automobile traffic. Indoor and outdoor concentrations of BTEX influenced lifetime cancer risk.
Vu, D. C., et al., (2019) [70]	USA	Cross-sectional	Children from four facilities of Head Start programs	Air pump. 2014.	VOCs	Human health risks associated with the targeted VOCs as measured by cancer risk	Sources of VOCs included vehicle-related emission, solvent-related emission, building materials, personal care products and household products. Potential carcinogenic compounds were benzene, ethylbenzene, naphthalene, 1,4-dichlorobenzene, tetrachloroethylene and trichloroethylene.
Vardoulakis, S., et al., (2020) [6]		Systemic review			VOC, PM_2.5_, NO_2_.		Household characteristics and occupant activities are essential in indoor exposure, especially cigarette smoking for PM_2.5_, gas appliances for NO_2_, and household products for VOCs and PAHs. Home location near high-traffic-density roads, redecoration, and small house size contribute to high indoor air pollution. High indoor particulate matter, NO_2_ and VOC levels were associated with respiratory symptoms, particularly asthma symptoms in children.
Weisel, C. P., et al., (2005) [71]	USA	Prospective cohort	100 non-smoking adult and children	Organic vapour monitor, personal environmental monitors	VOCs		The range of distribution for the VOCs, carbonyls, PM_2.5_, and air exchange rates, are consistent with values reported previously in the literature.
Weichenthal, S., et al., (2008) [72]		Review		Passive sampler	VOCs, UFP, NO_2_	Relationship between indoor nitrogen dioxide or VOC exposure and childhood asthma or related symptoms	VOC exposure have been more consistent in demonstrating a significant relationship with asthma or related symptoms.
Wangchuk, T., et al., (2015) [73]	Bhutan	Cross-sectional	82 children	Philips Aerasense NanoTracers, 2013.	UFP, VOCs, NO_2_		The highest UFP exposure resulted from cooking/eating, contributing to 64% of the daily exposure, resulting from firewood combustion in houses using traditional mud cookstoves.
Xia, X., et al., (2020) [74]	Hong Kong	Prospective cohort	20 COPD patients and 20 healthy participants	MicroPEM™ sensor. 2017–2018.	PM_2.5_	Effects on oxygen saturations in COPD and healthy participants	Short-term exposure to PM_2.5_ results in acute declines of SpO2 in 0–3 h, and then became insignificant at 0–12 h.
Yang, F. H., et al., (2019) [75]	Hong Kong	Prospective cohort	73 urban residents	Personal exposure kit. 2015–2016.	UFP, PM_2.5_, PM_10_		PM_2.5_ concentrations were lowest in office, whereas highest in outdoor activities.
Zhu, Y. F., et al., (2005) [76]	USA	Prospective cohort	4 two-bedroom apartments	Scanning mobility particle sizer, common switching manifold, 2003, 2004	UFP		Indoor to outdoor ratios for ultrafine particle number concentrations depended strongly on particle size and indoor ventilation mechanisms.
Zamora, M. L., et al., (2018) [77]	USA	Prospective cohort	17 pregnant women	Personal Environmental Monitor, 2015	PM_2.5_, black carbon, and nicotine		Cooking activities contributed significantly to the total PM_2.5_.
Zhang, L. J., et al., (2018) [78]	China	Prospective cohort	57 children	TSI DUST TRAKTM DRX sampler, real-time laser diode photometers, 2013.	PM_2.5_		Children personal exposure was mainly associated with ambient air conditions, height of the classroom, and transportation mode to school.
Zhou, Y., et al., (2020) [79]	China	Prospective cohort	26 students	Portable MicroAeth BC Monitor, Miniature Diffusion Size Classifier. 2016.	UFP and Black carbon		Average level of BC was higher in outdoor than the household and transport. Average level of UFP was higher in indoor than transport.
Zhou, H. C., et al., (2020) [80]	China	Prospective cohort	67 non-smoking healthy retirees	Micro-aethalometer AE51. 2018, 2019.	UFP and Black carbon		Ambient BC concentration, ambient temperature, humidity, education level and air purifier significantly impact personal BC exposure.
Zusman, M., et al., (2020) [81]	USA	Prospective cohort	2982 healthy smokers and non-smokers, COPD patients.	Ogawa passive samplers, Harvard Personal Environmental Monitor. 2014–2016.	PM_2.5_, NO_2_, NO_x_		Models using socioeconomic, meteorological, behavioral, residential, and ambient-pollutant concentration data obtained from questionnaires, direct observations, and measurements can facilitate exposure characterization of research cohorts with much less effort and expense than the monitoring of all participants.

## Supplementary Materials

The following are available online at https://www.mdpi.com/article/10.3390/ijerph19148752/s1, Table S1. Quality Report [15,16,17,18,19,20,21,22,23,24,25,26,27,28,29,30,31,32,33,34,35,36,37,38,39,40,41,42,43,44,45,46,47,48,49,50,51,52,53,54,55,56,57,58,59,60,61,62,63,64,65,66,67,68,69,70,71,73,74,75,76,77,78,80,81,83], Table S2. Joanna Briggs Institute Critical Appraisal Tools.

## Abbreviations

BC	black carbon
CO	carbon monoxide
FeNO	Fractional exhaled Nitric Oxide
FEV1	Force Expiratory Volume in 1 s
MMEF	Maximal Mid-Expiratory Flow
NO_2_	nitrogen dioxide
PEFR	Peak Expiratory Flow Rate
PMC	Particle Mass Concentration
PM	Particulate Matter
PM_0.1_	PM smaller than 0.1 microns
PM_1_	PM smaller than 1 micron
PM_2.5_	PM smaller than 2.5 microns
PM_10_	PM smaller than 10 microns
PNC	size-specific Particle Number Concentration
PRISMA	Preferred Reporting Items for Systematic reviews and Meta-Analyses
PSC	Particle Surface area Concentration
RCT	Randomized Control Trial
UFP	Ultrafine particles
WHO	World Health Organization
