# Supplementary material for: Indoor Air Pollution and the Health of Vulnerable Groups: A Systematic Review Focused on Particulate Matter (PM), Volatile Organic Compounds (VOCs) and Their Effects on Children and People with Pre-Existing Lung Disease
Source: Int J Environ Res Public Health. 2022 Jul 19;19(14):8752. doi: 10.3390/ijerph19148752 (PMC9316830; doi:10.3390/ijerph19148752)
Supplement: Supplementary file 1 [file ijerph-19-08752-s001.zip › ijerph-1689421-supplementary.pdf]

Supplementary Materials

Table S1. Quality Report (order in year of publication, TZM and EH).

	**1**	**2**	**3**	**4**	**5**	**6**	**7**	**8**	**9**	**10**	**11**	**Overall**
Raaschou-Nielsen, O. [59], et al., (1997)	H	H	H	L	H	H	H	H				H (Cross sectional)
Rojas-Bracho, L. [60], et al., (2000)	NA	H	H	H	H	H	H	H	U	U	H	H
Adgate, J. L. [15], et al., (2004)	L	H	H	L	H	H	H	H	U	U	H	H
Adgate, J. L. [16], et al., (2004)	L	H	H	L	H	H	H	H	U	U	H	H
Shendell, D. G. [63], et al., (2004)	L	H	H	L	L	H	H	H	U	U	H	L
Rojas-Bracho, L. [61], et al., (2004)	NA	H	H	H	H	H	H	H	U	U	H	H
Sexton, K. [64], et al., (2005)	L	H	H	H	H	H	H	H	U	U	H	H
Batterman, S. [17], et al (2005)	NA	H	H	H	H	H	H	H	H	H	H	H
Jansen, K. L. [44], et al., (2005)	NA	H	H	H	H	H	H	H	L	L	H	H
Weisel, C. P. [71], et al., (2005)	L	H	H	H	H	H	H	H	U	U	H	H
Zhu, Y. F. [76], et al., (2005)	NA	H	H	H	H	H	H	H	U	U	H	H
Phillips, M. L. [56], et al., (2005)	NA	H	H	H	H	H	H	H	U	U	H	H
Delfino, R. J. [32], et al., (2006)	L	H	H	H	H	H	H	H	U	U	H	H
Trenga, C. A. [68], et al., (2006)	NA	H	H	H	H	H	H	H	U	U	H	H
Diapouli, E. [33], et al., (2007)	H	H	H	H	U	U	H	U				H (Cross sectional)
Cortez-Lugo, M. [25], et al., (2008)	NA	H	H	H	H	H	H	H	H	H	H	H
Diapouli, E. [34], et al., (2008)	H	H	H	H	U	U	H	L				H (Cross sectional)
Gokhale, S. [37], et al., (2008)	L	H	H	L	L	H	H	H	H	NA	H	L
Goyal, R. and M. Khare [38] (2009)	NA	H	H	H	H	H	H	H	H	U	H	H
Sohn, H. and K. Lee [65] (2010)	NA	H	H	H	H	H	H	H	H	NA	H	H
Massolo, L. [50], et al., (2010)	NA	H	H	U	U	H	H	H	U	U	H	L
Byun, H. [18], et al., (2010)	L	H	H	U	H	H	H	H	U	U	H	H
Guo, H. [39], et al., (2010)	H	H	H	H	L	L	H	H				H
Kearney, J. [47], et al., (2011)	NA	H	H	H	H	H	H	H	H	H	H	H
Habil, M. and A. Treja [42] (2011)	H	H	H	H	H	U	L	H				H (Cross-sectional)
Broich, A. V. [19], et al., (2012)	L	H	H	L	U	H	H	H	H	H	H	H
Buonanno, G. [20], et al., (2012)	L	H	H	H	H	H	H	H	U	U	H	H
Buonanno, G. [21], et al., (2013)	L	H	H	H	H	H	H	H	U	U	H	H
Gao, Y. [40], et al., (2014)	H	H	H	H	H	H	H	H	H	H		H (Case Control)
Baumgartner, J. [22], et al., (2014)	L	H	H	H	H	H	H	H	H	H	H	H
Branco, P. [23], et al., (2014)	H	H	H	H	L	L	H	H				H (Cross sectional)
Mazaheri, M. [52], et al., (2014)	H	H	H	H	L	L	H	H				H (Cross sectional)
Beko, G. [24], et al., (2015)	H	H	H	H	H	H	H	H				H (Cross sectional)
Rufo, J. C. [62], et al., (2015)	H	H	H	H	H	H	H	H				H (Cross sectional)
Wangchuk, T. [73], et al., (2015)	H	H	H	H	H	H	H	H				H (Cross sectional)
Cortez-Lugo, M. [26], et al., (2015)	NA	H	H	L	U	H	H	H	H	H	H	H
Cipolla, M. [27], et al., (2016)	H	H	H	U	U	H	H	H	L	L	H	L
Mainka, A. and B. Kozielska [51] (2016)	H	H	H	H	H	H	H	H	H	U	H	H
Kalimeri, K. K. [48], et al., (2016)	H	H	H	H	H	H	L	H	U	U	H	H
Cleary, E. [28], et al., (2017)	H	H	H	H	L	L	U	H				H (Cross sectional)
Paunescu, A. C. [57], et al., (2017)	L	H	H	H	H	H	H	H	U	U	H	H
de Kluizenaar, Y. [83], et al., (2017)	NA	H	H	L	U	H	H	H	U	H	H	H
Hoang, T. [43], et al., (2017)	H	H	H	H	L	L	H	H				H (Cross sectional)
Jeong, H. and D. Park [45] (2017)	L	H	H	H	H	H	H	H	H	U	H	H
Zamora, M. L. [77], et al., (2018)	L	H	H	H	H	H	H	H	U	U	H	H
Zhang, L. J. [78], et al., (2018)	NA	H	H	H	H	H	H	H	L	U	H	H
Jeong, H. and D. Park [46] (2018)	NA	H	H	H	H	H	H	H	U	U	H	H
Cheung, P. K. [29], et al., (2019)	NA	H	H	H	H	H	H	H	U	U	H	H
Mazaheri, M. [53], et al., (2019)	NA	H	H	H	H	H	H	H	H	H	H	H
Slezakova, K. [67], et al., (2019)	H	H	H	H	H	H	H	H				H (Cross Sectional)
Yang, F. H. [75], et al., (2019)	L	H	H	L	L	H	H	H	H	H	H	H

Cunha-Lopes, I. [30], et al., (2019)	NA	H	H	L	L	H	H	H	L	L	H			L
Vu, D. C. [70], et al., (2019)	H	H	H	H	H	U	H	H						H (Cross sectional)
Curto, A. [31], et al., (2019)	L	H	H	U	U	H	H	H	H	H	H			H
Garcia-Hernandez, C. [41], et al., (2019)	H	H	H	H	H	U	U	H	L	H	H			H (Systemic Review)
Vardoulakis, S. [6], et al., (2020)	H	H	H	H	H	H	H	U	U	H	H			H (Systemic review)
Tran, T. D. [69], et al., (2020)	H	H	H	H	L	L	H	H						H(Cross sectional)
Liu, Y. W. [49], et al., (2020)	NA	H	H	L	L	H	H	H	U	L	H			L
Zhou, Y. F. [76], et al., (2020)	L	H	H	L	L	H	H	H	U	U	H			L
Zhou, H. C. [80], et al., (2020)	NA	H	H	H	H	H	H	H	H	H	H			H
Pacitto, A. [58], et al., (2020)	NA	H	H	U	U	H	H	H	U	U	H			L
Martins, V. [54], et al., (2020)	H	H	H	H	L	L	H	H						H (Cross sectional)
Faria, T. [36], et al., (2020)	H	H	H	H	L	L	H	H						H (Cross sectional)
Xia, X. [74], et al., (2020)	H	H	H	H	H	H	H	H	H	H	H			H
Zusman, M. [81], et al., (2020)	H	H	H	H	H	H	H	H	H	U	H			H
Martins, V. [55], et al., (2020)	H	H	H	H	L	L	H	H						H (Cross sectional)
**RCT**	**1**	**2**	**3**	**4**	**5**	**6**	**7**	**8**	**9**	**10**	**11**	**12**	**13**	**Overall**
Fang, L. [35], et al., (2019)	H	H	H	H	H	H	H	H	H	H	H	H	H	H
Soppa, V. J. [66], et al., (2014)	H	L	H	L	L	L	H	H	H	H	H	H	H	H

Judgement (H—High, L—Low, U—Unclear, NA—not applicable).

Table S2. Joanna Briggs Institute Critical Appraisal Tools.

	Cohort	Cross-Sectional	Case Control	RCT	Systemic Review
1	Were the two groups similar and recruited from the same population?	Were the criteria for inclusion in the sample clearly defined?	Were the groups comparable?	Was true randomization used for assignment of participants?	Is the review question clearly and explicitly stated?
2	Were the exposures measured similarly to both exposed and unexposed groups?	Were the study subjects and the setting described in detail?	Were cases and controls matched appropriately?	Was allocation to treatment groups concealed?	Were the inclusion criteria appropriate for the review question?
3	Was the exposure measured in a valid and reliable way?	Was the exposure measured in a valid and reliable way?	Were the same criteria used for identification of cases and controls?	Were treatment groups similar at the baseline?	Was the search strategy appropriate?
4	Were confounding factors identified?	Were objective, standard criteria used for measurement of the condition?	Was exposure measured in a standard, valid and reliable way?	Were participants blind to treatment assignment?	Were the sources and resources used to search for studies adequate?
5	Were strategies to deal with confounding factors stated?	Were confounding factors identified?	Was exposure measured in the same way for cases and controls?	Were those delivering treatment blind to treatment assignment?	Were the criteria for appraising studies appropriate?
6	Were the participants free of the outcome at the start of the study?	Were strategies to deal with confounding factors stated?	Were confounding factors identified?	Were outcomes assessors blind to treatment assignment?	Was critical appraisal conducted by two or more reviewers?
7	Were the outcomes measured in a valid and reliable way?	Were the outcomes measured in a valid and reliable way?	Were strategies to deal with confounding factors stated?	Were treatment groups treated identically?	Were there methods to minimize errors in data extraction?
8	Was the follow up time reported and sufficient to be long enough?	Was appropriate statistical analysis used?	Were outcomes assessed in a standard, valid and reliable way for cases and controls?	Was follow up complete and if not, were differences between groups in terms of their follow up adequately described and analyzed?	Were the methods used to combine studies appropriate?
9	Was follow up complete, and if not, were the reasons to loss to		Was the exposure period of interest long enough to be meaningful?	Were participants analyzed in the groups to which they were randomized?	Was the likelihood of publication bias assessed?

	follow up described and explored?				
10	Were strategies to address incomplete follow up utilized?		Was appropriate statistical analysis used?	Were outcomes measured in the same way for treatment groups?	Were recommendations for practice supported by the reported data?
11	Was appropriate statistical analysis used?			Were outcomes measured in a reliable way?	Were the specific directives for new research appropriate?
12				Was appropriate statistical analysis used?	
13				Was the trial design appropriate, and any deviations from the standard RCT design accounted for in the conduct and analysis of the trial?	
